# Supplementary material for: Deep learning prediction of noise-driven nonlinear instabilities in fibre optics
Source: Nat Commun. 2025 Aug 21;16:7800. doi: 10.1038/s41467-025-62713-x (PMC12371006; doi:10.1038/s41467-025-62713-x)
Supplement: Supplementary file 1 — Supplementary Information [file 41467_2025_62713_MOESM1_ESM.pdf]

Supplementary Information

Deep learning prediction of noise-driven nonlinear instabilities in fibre optics

Yassin BOUSSAFA[1], Lynn SADER[1], Van Thuy HOANG[1], Bruno P. CHAVES[1], Alexis BOUGAUD[1], Marc FABERT[1], Alessandro TONELLO[1], John M. DUDLEY[2,3], Michael KUES[4], Benjamin WETZEL[1,*]

[1] XLIM Research Institute, CNRS UMR 7252, University of Limoges, 87060, Limoges, France
[2] Université Marie et Louis Pasteur, CNRS Institut FEMTO-ST, 25030, Besançon, France
[3] Institut Universitaire de France, Paris, France
[4] Institute of Photonics and Cluster of Excellence PhoenixD, Leibniz University Hannover, 30167, Hannover, Germany
**benjamin.wetzel@xlim.fr*

On the advantages of machine learning schemes compared to classical techniques

We provide below a summary table that succinctly summarizes the benefits and limitations of machine learning (ML) approaches compared to traditional numerical and signal processing methods in the framework of nonlinear fibre optics and the study modulation instability (MI). Table S1 is here only provided as a general guide for the reader aiming to have a broad view on the current impact of ML on this particular applicative research field. For a more detailed and referenced comparison of the advantages and limitations of ML compared to standard numerical modelling or classical signal processing techniques, one can refer to recent review articles on the topic[1–7].

**Methods**	**Description**	**Advantages (+) and Limitations (-)**
**Modeling nonlinear pulse evolution in guided wave optics**		
Analytic & Theory	Variety of NLSE-based approaches and simplified models[8]	+ Physical insight into the underlying propagation mechanisms.<br>+ Potential normalization/generalization.<br>- Limited validity to specific approximations & particular dynamics.
Numerical	Numerical integration of the GNLSE[9]	+ Reliable workhorse with excellent accuracy.<br>+ Multiple Monte-Carlo realizations typically used for modeling incoherent pulse propagation.<br>- Potentially slow integration (~100 ms- minutes).
ML-based	ANN-based integration of ultrashort pulse propagation[10–13]	+ Variety of neural networks (FFNN, LTSM) demonstrated for fast GNLSE-like integration, with reliable accuracy for most applications.<br>- Requires training on extensive dataset. Limited generalization.<br>- Typically limited to coherent pulse evolution.
ML-based	ANN-based transfer functions[14,15]	+ Access to key propagation characteristics.<br>+ Potential normalization & generalization.<br>- Requires training on quality datasets; Usually implemented in specific propagation regimes.<br>- Potentially sensitive to noise & limited to coherent pulse evolution.
**Design & control of nonlinear propagation dynamics**		
Theory & Simulations	Design of propagation dynamics (waveguide or pulse properties) based on theory or simulations	+ Fast parameter scan (theory); Reliable parameter scan (simulations)<br>- No guaranteed selection of optimal parameter<br>- Highly sensitive to experimental conditions (e.g. uncertainties on pulse properties /waveguide fabrication errors): Potentially limited practical applicability
Classical experimental techniques	Standard tuning of experimental parameters (e.g. pulse shaping, power adjustment, etc.)	+ Typically informed by theory and/or simulations.<br>+ Practical reconfigurability enabled by available experimental tools.<br>- Requires parameter scan (extensive sweep, Monte-Carlo methods).<br>- No guaranteed optimal.
ML-based	Metaheuristics approaches with e.g. tunable pulse reshaping	+ Optimization of nonlinear broadening and fibre propagation dynamics: Supercontinuum[16–19], frequency combs[20,21], laser cavity operations[22–25], telecom[7] & optical networks[6].<br>+ Robust to unstable and variable experimental conditions.<br>- Potentially slow convergence. No straightforward generalization nor ensured optimal.<br>- Requires new optimization for each individual target optimization.
ML-based	Inverse design[2] & deep-learning strategies[4]	+ Access to non-trivial waveguide/cavity/propagation designs[26–30] & pulse shaping[31].<br>- Highly sensitive to experimental conditions.
**Study, control & optimization of modulation instability (MI)**		
Analytical	Three-wave truncation, Akhmediev breather theory, Inverse Scattering Transform (IST), …	+ Validated theoretical frameworks & well-known solutions for describing MI dynamics[32–40]<br>- Inherent approximations.<br>- Complex description of noise-driven and weakly-seeded MI dynamics[41–44].<br>- Requires excellent knowledge of noise properties for incoherent MI study (via e.g. IST).
Numerical	Numerical integration of the GNLSE[9]	+ Reliable simulations with excellent accuracy (but slow integration).<br>- Multiple Monte-Carlo realizations for modeling incoherent pulse propagation.<br>- Requires excellent knowledge of noise properties for incoherent MI study.
Numerical & Experimental	Control of MI dynamics[45–50]	+ Variety of MI dynamics adjustment techniques (optical seeding, pulse shaping/ modulation,)<br>+ Control techniques transferable to ML-based optimization strategies (ANN, GA, PSO, etc.)<br>- Typically implemented on coherent processes
ML-based	ML-based MI control in fibre optics	+ Exploration of ideal four-wave mixing (FWM) dynamics and phase space topology[51].<br>+ Potential for generalization and physics-informed ML techniques (data-driven[52]).<br>- Limited to study and control of coherent MI processes.<br>- Limited to numerical studies[53].
**Incoherent signal characterization & instability forecast**		
Experimental (standard schemes)	Averaged spectral measurements (OSA)	+ High resolution & high dynamic range spectral measurements<br>- Averaged measurements: No access to-shot-to-shot incoherent spectra<br>- Intensity-data only (no phase)
Experimental (real-time schemes)	Real-time measurements techniques[54–58]	+ Access to single-shot properties via dispersive Fourier transform, time-lens/microscopy…<br>- Intensity-data only (no phase); Phase retrieval configurations available (additional complexity)<br>- Costly & high-bandwidth characterization systems.<br>- Limited dynamic range and resolution.
ML-based	ANN processing of incoherent signals[59–61]	+ Fourier-Transform like processing of single shot spectra (requires real-time measurements)<br>+ Prediction of temporal peak powers (from spectral intensity-data only).<br>+ Potential statistical analysis (e.g. extreme event occurrence, etc.)<br>- Requires extended and high dynamic-range data for ANN training.<br>- No active control of incoherent MI processes.
ML-based	ANN forecast of incoherent dynamics[62,63]	+ Extreme event forecast from time-series (e.g. in resonator/cavities).<br>+ Compatible with incoherent and chaotic signal forecast from precursors (via e.g. LSTM)<br>- Requires long-range (extended) and real-time data acquisition

Table S1: Overview of main advantages (+) and limitations (-) of machine-learning approaches compared to traditional numerical and signal processing methods in the area of nonlinear fibre optics and modulation instability processes.

Impact of coherent optical seeding on the stability of noise-driven modulation instability

As discussed in the main manuscript, we note that the spectral location (and phase) of the coherent seeds copropagating with noise in the highly nonlinear fibre (HNLF) have a noticeable impact on the resulting spectral broadening arising from concurrent modulation instability (MI) processes.

In our case, coherent optical seeding leads to a relative stabilization of the spectral fluctuations in particular spectral regions of the broadband output MI spectra. From our simulations, such spectral regions are featured with a coefficient of variation typically decreasing below 100 % (i.e. a standard deviation of the intensity fluctuations below the average intensity). Two illustrative examples studying such shot-to-shot spectral fluctuations are presented in Fig. S1, using two coherent seeds at the input with different properties.

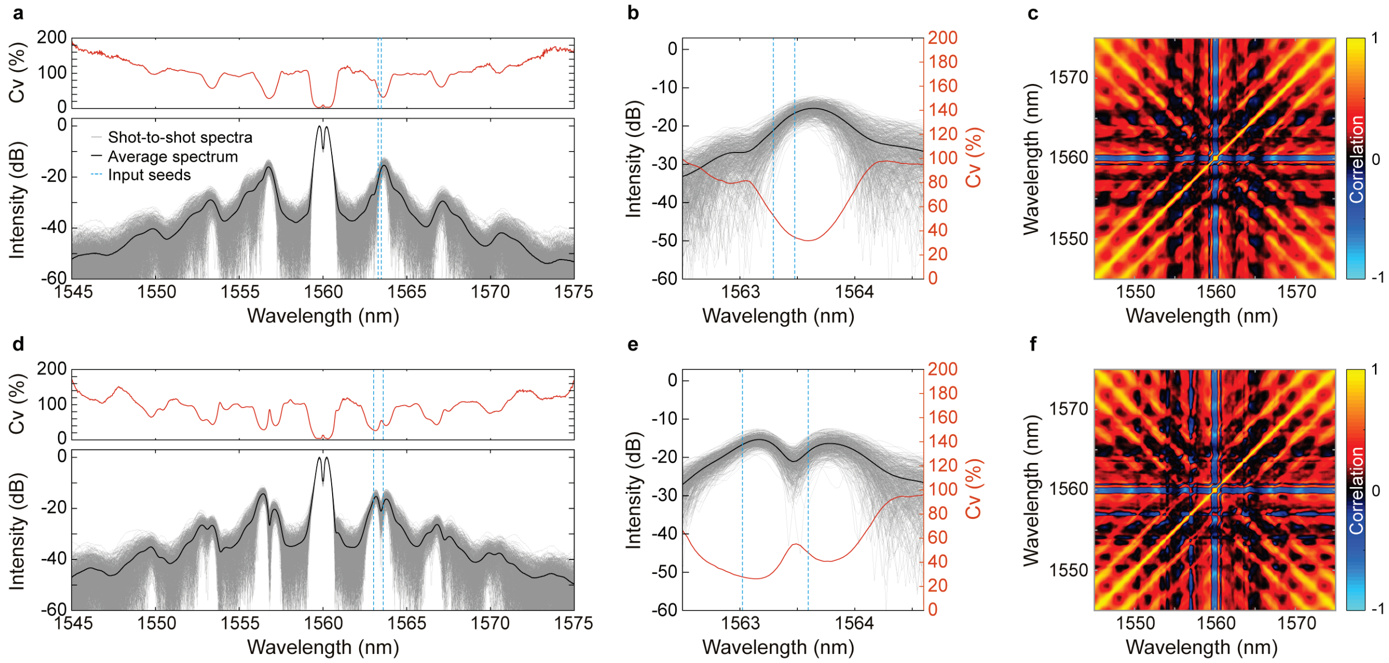

Figure S1: Illustration of the impact of coherent MI seeding on resulting output spectral fluctuations – 2 seed case. a. *Example of shot-to-shot spectral fluctuations obtained via numerical simulations (GNLSE) considering HNLF propagation for a selected two-seed configuration at the fibre input. Bottom: For this seeding configuration (input seed locations shown with dashed blue lines), the results of 200 different noisy realizations are shown with thin grey lines while the average spectrum is shown with a black line. Top: The corresponding coefficient of variation (C_v) of these spectral fluctuations, computed from noisy realizations, is displayed in red.* ***b.*** *Zoom on the output spectral fluctuations and the corresponding coefficient of variation around the input seed locations.* ***c.*** *Correlation map of the output spectrum shot-to-shot fluctuations computed from the different noisy realizations.* ***d-f****. Same as panels a-c, but considering another example of seeding conditions (see dashed blue lines for input seed locations).*

By analysing the output fluctuation properties with respect to the input seed locations, one can observe that these fluctuations remain significant (~10 dB or above) at the seeding wavelength (see dashed blue lines). More importantly, the seed locations do not specifically correspond to the point of maximal spectral intensity in the MI wings of the spectrum, nor the point where the relative fluctuations are the lowest (i.e. minimal coefficient of variation C_v). Yet, in such a case, the profiles of the average spectrum and corresponding correlation maps are modified by optical seeding.

This observation remains valid when considering a larger number of seeds (and thus a larger parameter space and enhanced complexity of the system). Fig. S2 provide similar illustrative examples of shot-to-shot spectral fluctuations observed when using four coherent input seeds with different properties.

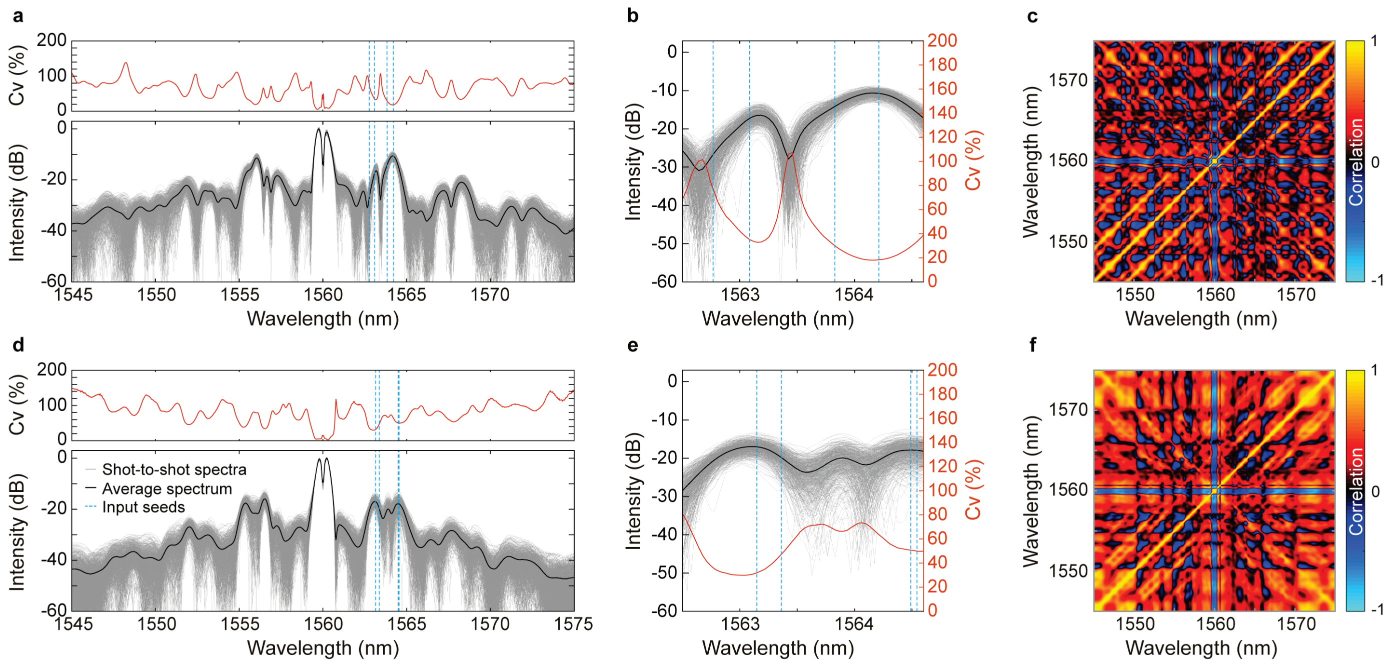

Figure S2: Illustration of the impact of coherent MI seeding on resulting output spectral fluctuations – 4 seed case. ***a.*** *Example of shot-to-shot spectral fluctuations obtained via numerical simulations (GNLSE) considering HNLF propagation for a selected two-seed configuration at the fibre input. Bottom: For this seeding configuration (input seed locations shown with dashed blue lines), the results of 200 different noisy realizations are shown with thin grey lines while the average spectrum is shown with a black line. Top: The corresponding coefficient of variation (C_v) of these spectral fluctuations, computed from noisy realizations, is displayed in red.* ***b.*** *Zoom on the output spectral fluctuations and the corresponding coefficient of variation around the input seed locations.* ***c.*** *Correlation map of the output spectrum shot-to-shot fluctuations computed from the different noisy realizations.* ***d-f****. Same as panels a-c, but considering another example of seeding conditions (see dashed blue lines for input seed locations).*

Similar to Fig. S1, one can see that there is no direct and trivial link between the input seed locations and the points of maximal intensity or stability in the output spectrum (or, conversely, the regions of maximum correlation or anticorrelation in the spectral correlation maps). This observation is significant, as it highlights the inherent complexity of the nonlinear dynamics at play. It also provides further insight in the unicity and diversity of the system. These examples indeed illustrate how the inference of the input seed parameters is not straightforward, especially when one increases the dimensionality of the parameters space (i.e. the number of seeds). However, an increased number of seeds seems to yield more specific signatures and features in the output spectrum and correlation map that thus leads to more robust ANN predictions, even in noisy experimental conditions (see e.g. Table S2 and S3).

Discussion on the long-term stability of experimental acquisitions

In the manuscript, we discussed the impact of experimental conditions and overall stability for the acquisition of reliable dataset for ANN training. Among other, we noticed that despite very good overall accuracy, a degradation of the ANN prediction of output features was observed in experiments when training the ANN from input seed conditions (rather than average output spectral measurements). Importantly, during our experiments, we monitored the overall stability of the system and DFT acquisition by periodically recoding the output features of a selected set of input seeding conditions. Every ~ 30 minutes (i.e. every 500 different Monte-Carlo seeding conditions were experimentally measured for ANN training), we performed a full check of the OSA and DFT spectra for an ensemble of 20 predefined seeding conditions - acting as a benchmark for our system stability. The results of such an experimental monitoring is presented in Fig. S3, summarizing the periodic measurements performed over > 60h for a specific seeding condition:

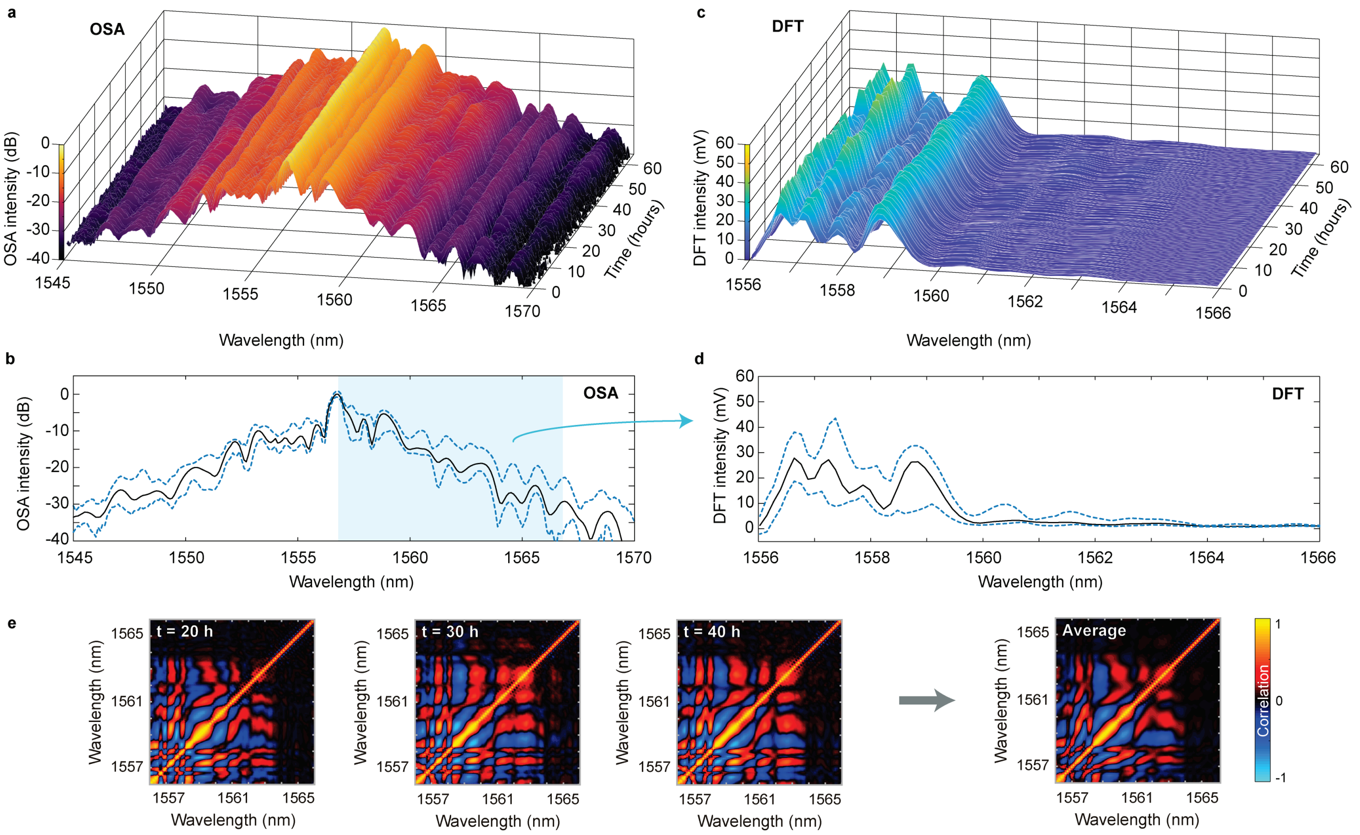

Figure S3: Long-term experimental stability during extended DFT dataset acquisition – Seeding configuration #1. a. *Sequential acquisition of OSA spectra, measured periodically for a given seeding configuration. The measurements are performed every ~30 minutes, interleaved for a period of >60 hours during the experimental Monte-Carlo dataset acquisition used for ANN training.* ***b.*** *The corresponding average spectrum from these periodic OSA measurements are shown with a black line, while the maximal deviation from the average spectrum during this periodic monitoring is shown with dashed blue lines. Blue shades correspond to the spectral region of interest where the spectrum is filtered for DFT acquisition.* ***c.*** *Sequential acquisition of DFT spectra, performed with the same periodicity as in panel a. Each average DFT spectrum is respectively averaged from 500 DFT acquisitions, similar to the measurements performed for statistical analysis and ANN training.* ***d.*** *Corresponding DFT average spectrum (black line) and maximal deviation (dashed blue lines) obtained from this periodic monitoring.* ***e.*** *Examples of spectral correlation map, extracted from DFT measurements performed at different times of this stability monitoring (after 20, 30 and 40 hours, respectively). The corresponding average correlation maps for these > 120 DFT measurements is also provided for completeness.*

From these acquisitions, we have observed a weak but noticeable long-term experimental instability in the system. For instance, in Fig. S3, both OSA and DFT spectral measurements present a drift and/or periodic modulation of the recorded spectral features along time. A very similar trend is also noticeable in Fig. S4, depicting the same periodic measurements performed for another seeding conditions in our benchmark.

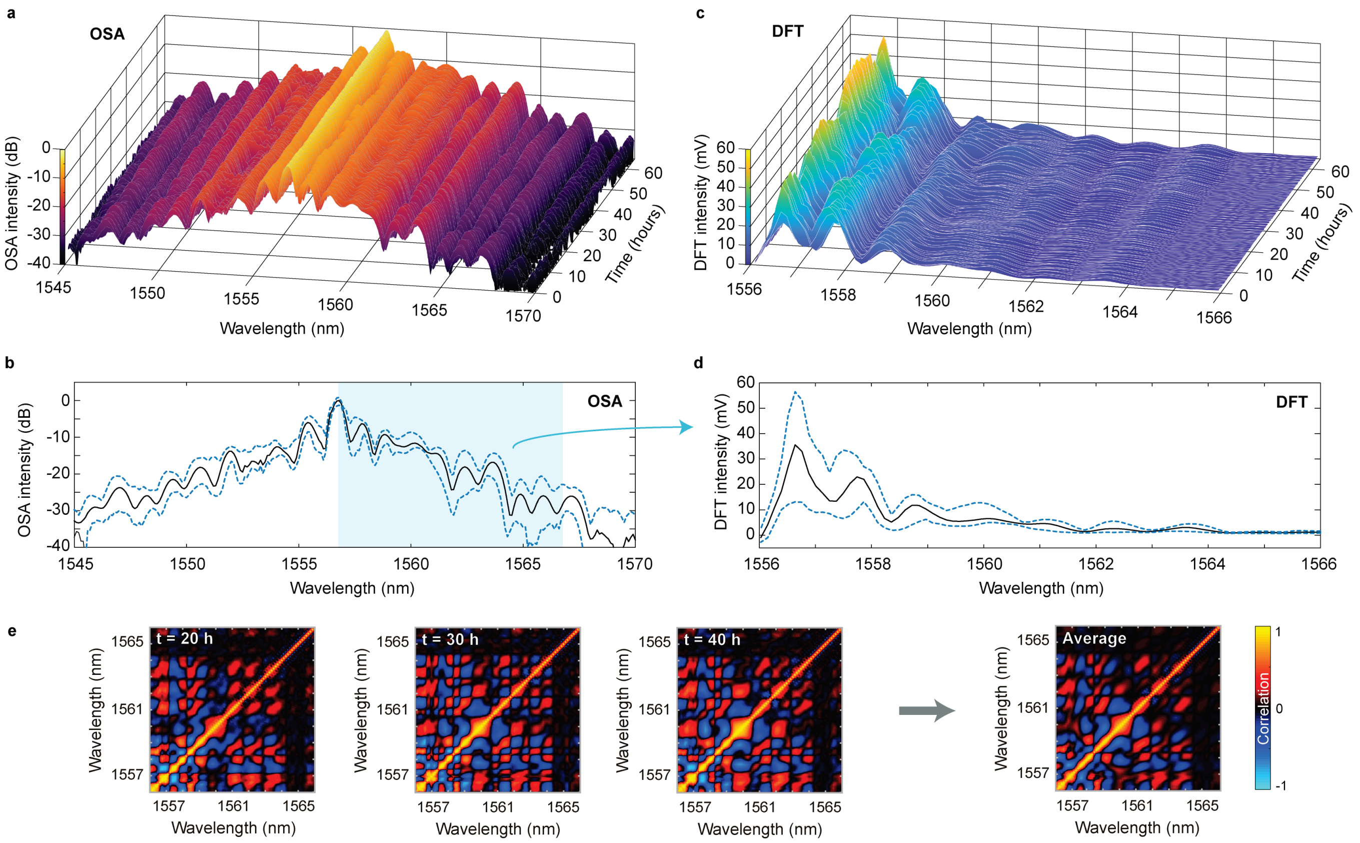

Figure S4: Long-term experimental stability during extended DFT dataset acquisition – Seeding configuration #2. *Same data as those presented in Fig. S3, but obtained from monitoring the stability of another seeding configuration:* ***a.*** *Sequential acquisition of OSA spectra, measured periodically for a given seeding configuration. The measurements are performed every ~30 minutes, interleaved for a period of >60 hours during the experimental Monte-Carlo dataset acquisition used for ANN training.* ***b.*** *The corresponding average spectrum from these periodic OSA measurements are shown with a black line, while the maximal deviation from the average spectrum during this periodic monitoring is shown with dashed blue lines. Blue shades correspond to the spectral region of interest where the spectrum is filtered for DFT acquisition.* ***c.*** *Sequential acquisition of DFT spectra, performed with the same periodicity as in panel a. Each average DFT spectrum is respectively averaged from 500 DFT acquisitions, similar to the measurements performed for statistical analysis and ANN training.* ***d.*** *Corresponding DFT average spectrum (black line) and maximal deviation (dashed blue lines) obtained from this periodic monitoring.* ***e.*** *Examples of spectral correlation map, extracted from DFT measurements performed at different times of this stability monitoring (after 20, 30 and 40 hours, respectively). The corresponding average correlation maps for these > 120 DFT measurements is also provided for completeness.*

Altogether, these results show that the detected long-term instability in the setup (shown in Fig. 4 of the manuscript) can be attributed to the erbium-doped fibre amplifier (EDFA) rather than the programmable filter (i.e. Waveshaper) repeatability. Indeed, EDFA amplification is highly sensitive to thermal fluctuations. In particular, in Figs. S3 and S4 (see panels a and b), one can notice a close to 24h period that can be attributed to thermal cycling within the lab (not perfectly compensated by air conditioning regulation). As a consequence, reshaping of the output spectra can be observed along the 60+ hours of monitoring. More importantly, while the average spectral correlation maps maintain the same predominant features over time, one can see that the periodic recording of these correlation maps exhibit minor discrepancies throughout time (see in panels e in Figs. S3 and S4).

As such, these observations can readily explain the limited accuracy of the experimentally-trained ANN in the forecast of the output spectrum and spectral correlation maps from the seed parameters (see Figs. 5 and 6 as well as Table S3). The main spectral features are correctly predicted but limited long-term stability hampers the prediction of finer structures, in contrast the numerical observations or the use of the output average spectrum to predict the correlation features (with an ANN trained on dataset acquired while experiencing such thermal drifts). Importantly, this shows that the prediction of correlation features is robust to the EDFA fluctuations, which are somehow incorporated within the ANN training performed from the output data.

Results overview of neural network prediction and inference

For completeness, we provide below a summary of the trained ANN prediction accuracy. The results obtained from ANNs trained with data generated via numerical simulations (based on the GNLSE) are shown in Table S2. Herein, we compare the predictions results for different variables (i.e. seed properties, average spectrum, correlation maps) obtained when either considering two or four input seeds with tunable wavelength and phase, as discussed in the main manuscript. Besides the normalized ANN error provided in terms of RMSE, we also show the corresponding error in physical units to illustrate the overall accuracy of the ANN predictions.

Numerical data	2 seed input		4 seed input	
	RMS error	Physical error	RMS error	Physical error
Seed wavelength	2.1 %	44 pm	5.0 %	105 pm
Seed phase	3.1 %	5.6 °	8.6 %	15.5 °
Average spectrum	1.1 %	0.6 dB	2.9 %	1.45 dB
Correlation map	3.7 %	//	7.4 %	//

Table S2: Summary of ANN prediction accuracy when trained from numerically-simulated data.

Similarly, the results obtained from ANNs trained with experimental data are provided in Table S3:

Experimental data	2 seed input		4 seed input	
	RMS error	Physical error	RMS error	Physical error
Seed wavelength	4.9 %	245 pm	15.4 %	770 pm
Seed phase	7.3 %	13.1 °	24.2 %	43.5 °
Average spectrum (ANN trained from correlation maps)	0.9 %	//	0.8 %	//
Average spectrum (ANN trained from seed parameters)	4.1 %	//	2.6 %	//
Correlation map (ANN trained from average spectrum)	4.0 %	//	3.9 %	//
Correlation map (ANN trained from seed parameters)	8.1 %	//	7.6 %	//

Table S3: Summary of ANN prediction accuracy when trained from experimentally-measured data.

Neural network architecture & training parameters

For completeness, we provide below a summary of the ANN architectures (Table S4) and training parameters (Table S5) used for the prediction and inference results reported in the main manuscript. In both tables, we explicitly mention in which figure the results obtained from ANN training are shown in the main manuscript (i.e. manuscript reference).

As seen in Table S4, for all dataset and corresponding ANN trainings, the neural network used corresponds to a fully connected feed-forward network (FFNN) of constant depth (5 layers in total), comprising 1 input layer, 3 (hidden) inner layers, and 1 output layer. The type of input and output data used for ANN training (i.e. inference of input seed parameters, or prediction of output spectra or correlation maps) thus conditions the respective number of perceptrons in the input/output layers. The key difference between different neural network architectures thus corresponds to adjustment of the respective widths (i.e. number of perceptrons per layer) of the layers within the FFNN depending on the data type and vector size used for training.

ID	Selected dataset for ANN training		Manuscript reference	Input & output data used ANN training		ANN architecture: FFNN (number of perceptrons per layer)					Total ANN perceptrons
	Type	*Seeds*		*Input*	*Output*	*Input*	*Inner #1*	*Inner #2*	*Inner #3*	*Output*	
**1**	Num	2	Fig. 6a	Seed parameters	Correlation maps	**4**	32	256	1024	**16384**	**17700**
**2**	Num	2	Fig. 3a	Seed parameters	Output spectra	**4**	8	128	512	**1024**	**1676**
**3**	Num	2	Fig. 3b	Correlation maps	Seed parameters	**16384**	1024	256	32	**4**	**17700**
**4**	Num	4	Fig. 6b	Seed parameters	Correlation maps	**16**	32	256	1024	**16384**	**17712**
**5**	Num	4	Fig. 3c	Seed parameters	Output spectra	**16**	32	128	512	**1024**	**1712**
**6**	Num	4	Fig. 3d	Correlation maps	Seed parameters	**16384**	16384	4096	512	**16**	**37392**
**7**	Exp	2	Fig. 7a *(bottom panel)*	Seed parameters	Correlation maps	**4**	1024	4096	16384	**6724**	**28232**
**8**	Exp	2	Fig. 5a *(orange dashes)*	Seed parameters	Output spectra	**4**	4096	512	256	**82**	**4950**
**9**	Exp	2	Fig. 5b	Correlation maps	Seed parameters	**6724**	16384	4096	1024	**4**	**28232**
**10**	Exp	2	Fig. 5a *(blue line)*	Correlation maps	Output spectra	**6724**	4096	512	256	**82**	**11670**
**11**	Exp	2	Fig. 7a *(middle panel)*	Output spectra	Correlation maps	**82**	1024	4096	16384	**6724**	**28310**
**12**	Exp	4	Fig. 7b *(bottom panel)*	Seed parameters	Correlation maps	**16**	1024	4096	16384	**6724**	**28244**
**13**	Exp	4	Fig. 5c *(orange dashes)*	Seed parameters	Output spectra	**16**	512	2048	8192	**82**	**10850**
**14**	Exp	4	Fig. 5d	Correlation maps	Seed parameters	**6724**	16384	4096	512	**16**	**27732**
**15**	Exp	4	Fig. 5c *(blue line)*	Correlation maps	Output spectra	**6724**	16384	4096	1024	**82**	**28310**
**16**	Exp	4	Fig. 7b *(middle panel)*	Output spectra	Correlation maps	**82**	1024	4096	16384	**6724**	**28310**

Table S4: Summary of ANN architectures and dataset used for the different prediction and inference results reported in the main manuscript (i.e. manuscript reference in column 4). *(Num = Numerical dataset, Exp= Experimental dataset, FFNN = Fully-connected Feed-Forward Neural Network).*

Depending on the specific data used (see Table S4), yielding modifications of the width and overall number of perceptron in the respective FFNN reported (ranging from 1676 up to 37392), slight empirical adjustments were made in the training hyperparameters from one case to another. For instance, marginal tuning of the learning rate (between 0.015 and 0.018) as well

as the maximal number of epochs (between 100 and 700) was made to ensure training accuracy and stability while avoiding unnecessary computation time. A summary of these hyperparameters for each case reported in the manuscript is further provided in Table S5. In this case, we also report the corresponding training time of each network, performed on *Matlab* (*Deep Learning* toolbox – SGDM solver) via the graphical processing units (GPU) of either of our workstations (both equipped with a Nvidia RTX 3090 featuring 24 GB of RAM). We note that these training times are indicative and may be further reduced by fine-tuning the network architectures and hyperparameters deepening the dataset (see e.g. halted training in Table S5 for a reduced number of epochs due to marginal training improvements). For instance, using a simpler architecture (i.e. 17236 perceptrons), training #14 could be achieved in less than 45 mins with less than 400 epochs to reach a similar RMSE in the predictions (i.e. 15.6% in the wavelength and 24.3% in the phase inference of the seeds, respectively).

ID	Selected dataset for ANN training		Manuscript reference	Input & output data used ANN training		Total ANN perceptrons	ANN learning rate	ANN training epochs	Computer specifications	ANN training runtime *(avg/epoch)*	ANN training runtime *(Total)*
	Type	*Seeds*		*Input*	*Output*						
**1**	Num	2	Fig. 6a	Seed parameters	Correlation maps	**17700**	0.018	215/300 *(halted)*	Workstation #1 with RTX 3090	43.9 s	157 min
**2**	Num	2	Fig. 3a	Seed parameters	Output spectra	**1676**	0.015	300	Workstation #1 with RTX 3090	20.0 s	99 min
**3**	Num	2	Fig. 3b	Correlation maps	Seed parameters	**17700**	0.017	500	Workstation #1 with RTX 3090	28.7 s	238 min
**4**	Num	4	Fig. 6b	Seed parameters	Correlation maps	**17712**	0.017	151/300 *(halted)*	Workstation #1 with RTX 3090	57.3 s	144 min
**5**	Num	4	Fig. 3c	Seed parameters	Output spectra	**1712**	0.018	100	Workstation #1 with RTX 3090	23.8 s	39 min
**6**	Num	4	Fig. 3d	Correlation maps	Seed parameters	**37392**	0.017	700	Workstation #1 with RTX 3090	49.5 s	577 min
**7**	Exp	2	Fig. 7a *(bottom panel)*	Seed parameters	Correlation maps	**28232**	0.015	300	Workstation #2 with RTX 3090	24.3 s	121 min
**8**	Exp	2	Fig. 5a *(orange dashes)*	Seed parameters	Output spectra	**4950**	0.017	500	Workstation #1 with RTX 3090	12.4 s	103 min
**9**	Exp	2	Fig. 5b	Correlation maps	Seed parameters	**28232**	0.015	700	Workstation #2 with RTX 3090	20.0 s	233 min
**10**	Exp	2	Fig. 5a *(blue line)*	Correlation maps	Output spectra	**11670**	0.017	500	Workstation #1 with RTX 3090	15.7 s	131 min
**11**	Exp	2	Fig. 7a *(middle panel)*	Output spectra	Correlation maps	**28310**	0.015	232/300 *(halted)*	Workstation #2 with RTX 3090	23.3 s	90 min
**12**	Exp	4	Fig. 7b *(bottom panel)*	Seed parameters	Correlation maps	**28244**	0.015	700	Workstation #2 with RTX 3090	21.3 s	248 min
**13**	Exp	4	Fig. 5c *(orange dashes)*	Seed parameters	Output spectra	**10850**	0.017	500	Workstation #2 with RTX 3090	12.1 s	101 min
**14**	Exp	4	Fig. 5d	Correlation maps	Seed parameters	**27732**	0.016	700	Workstation #2 with RTX 3090	19.1 s	222 min
**15**	Exp	4	Fig. 5c *(blue line)*	Correlation maps	Output spectra	**28310**	0.017	500	Workstation #2 with RTX 3090	19.2 s	160 min
**16**	Exp	4	Fig. 7b *(middle panel)*	Output spectra	Correlation maps	**28310**	0.015	700	Workstation #2 with RTX 3090	21.4 s	251 min

Table S5: Summary of ANN trainings for the different prediction and inference results reported in the main manuscript. *The dataset used and ANN architectures used correspond to the ID provided in Table S4. ANN trainings were performed via Graphical processing Units (GPU), on two different workstations, both equipped with the same Nvidia RTX 3090 (24 GB). The two workstations have different hardware configurations, however providing similar performance for such ANN trainings with the same GPU (RTX 3090). Workstation #1: Intel Core i9 10900 (10 cores - 2.8/5.2 GHz) with 64 GB RAM. Workstation #2: AMD Ryzen 9 5950X (16 cores – 3.4/4.9 GHz) with 128 GB RAM. In some cases, in the absence of noticeable ANN performance improvements, the training was halted before the end of the maximal number of epochs selected.*

On-demand tailoring of spectral fluctuation via ANNs – Numerical proof-of-concept

To conclude our study, we further illustrate the potential of the previously trained neural networks for the rapid generation of a large dataset, so that to optimize particular correlation features in incoherent MI spectra. To this end, we performed a proof-of-concept demonstration via a numerical study which principle is summarized in Fig. S5.

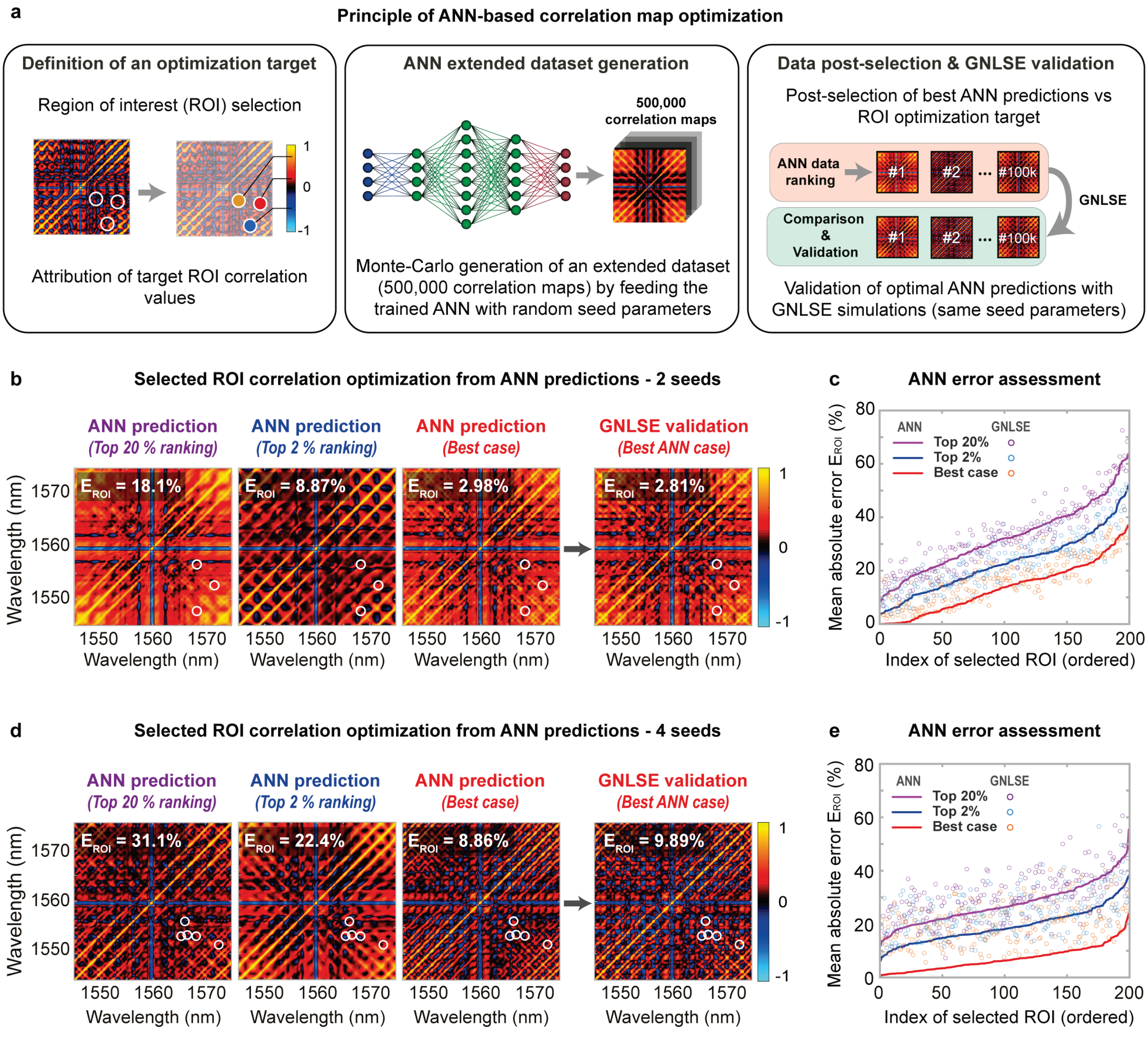

__Figure S5:__ ***Principle and numerical results for on-demand ANN-based spectral correlation map optimization.*** ***a.*** *Illustrative scheme of the method used for on-demand correlation map optimization via an ANN strategy. An arbitrary region of interest (ROI) and associated correlation values are first selected as an optimization target. The trained ANN is then leveraged to quickly search the parameters space by generating correlation maps for random seeding conditions. The ANN results are then ranked and the best scenarios are selected for numerical validation via GNLSE simulations.* ***b.*** *Example of ANN-based results using 2 adjustable input seeds for the optimization of a target ROI with 3 points: A selection of correlation maps generated from the trained network (ANN prediction) and their corresponding error for the selected ROI (E_{ROI}) are displayed based on their ranking. We show the maps ranked within the best 20%, the best 2%, and the absolute best case within the 500,000 ANN-generated maps. For the best ANN case, showing a predicted 2.98 % error, the corresponding map obtained from GNLSE simulations using the same seeding conditions is displayed (with an error E_{ROI} = 2.81 %).* ***c.*** *Summary of ANN-based optimization results for 200 different ROI randomly selected within the correlation maps. For each ROI, a comparison of the error obtained from ANN-generated maps (solid lines) and GNLSE-simulated maps (circles) is provided. For clarity, the ROI are sorted based on the quality of ANN predictions. The results are also color-coded depending on the ranking of ANN-generated maps (i.e. top 20 %, top 2%, absolute best).* ***d-e****. Same results as panels b-c, but obtained considering the case of 4 adjustable input seeds for the optimization of a target ROI with 5 points in the output spectral correlation maps.*

First, we selected a set of 200 random regions of interest (ROI) in the spectral correlation maps (i.e. 3 or 5 random pixels, corresponding to three or five pairs of wavelengths in the map). For each ROI pixel, we attributed a random correlation value. Each ROI (and associated correlations values) then become an optimization target for the trained neural network.

Second, we generated 500,000 random seed configurations. We then used the neural network trained on numerical data to predict each of the correlation map resulting from these seed configurations (see Fig. 6). This approach enables us to generate a substantial dataset in 2 minutes, which is approximately 50,000 times faster than using GNLSE-based numerical simulations.

Subsequently, we used the ANN-generated dataset to conduct stochastic optimization (i.e. post-selection) on the correlation values within the correlation maps. This process allows us to identify seed configurations that produce correlation maps with signature features that matches the best the desired optimization target (i.e. previously selected ROI correlation values).

Finally, to validate the network's capacity in predicting relevant correlation features, we calculated the correlation map from the selected seed configuration(s) via GNLSE simulations that we compare with the ones generated by the ANN.

The results provided in Fig. S5 clearly illustrate the efficiency of suitably post-selected ANN predictions for tailoring a signal with the targeted correlation features. For instance, in Fig. S5b, the agreement with ANN-generated maps and the GNLSE simulations is excellent. In both cases, the selected dual seeding scenario provide a great fit with the ROI targeted correlation values – yielding less than 3% average error (E_{ROI}, averaged over the 3 ROI pixels). A similar result is illustrated in Fig. S5d, with an error of E_{ROI} ~ 9 % for both ANN and GNLSE cases and the targeted optimization of 5 correlation map pixels via 4 suitably selected input optical seeds.

It is important to note that, following this framework, the quality of the fit is typically limited by the error inherent to the neural network after training (i.e. RMSE in the correlation map prediction). This feature is illustrated in Fig. S5c and e, comparing ANN and GLSE predictions for the 200 targeted ROI. Importantly, the agreement between ANN and GNLSE remains good, although some discrepancies can appear for the very best ANN predictions (see Fig. S5e). This property is actually due to the fact that the ROI targeted optimization is here randomly selected, and may actually not correspond to correlation features physically achievable by the system. In this framework, and although this falls outside of the scope of this study, we expect that a thorough statistical analysis of the data produced by the ANN (e.g. variations and statistical distribution of the error E_{ROI} within the top ANN-generated maps) might allow gaining insight in the existence of a suitable and physical solution within the accessible parameter space.

References

1. Shastri, B. J. *et al.* Photonics for artificial intelligence and neuromorphic computing. *Nat. Photonics* **15**, 102–114 (2021).
2. Mengu, D. *et al.* At the intersection of optics and deep learning: statistical inference, computing, and inverse design. *Adv. Opt. Photonics* **14**, 209–290 (2022).
3. Genty, G. *et al.* Machine learning and applications in ultrafast photonics. *Nat. Photonics* **15**, 91–101 (2021).
4. Freire, P., Manuylovich, E., Prilepsky, J. E. & Turitsyn, S. K. Artificial neural networks for photonic applications—from algorithms to implementation: tutorial. *Adv. Opt. Photonics* **15**, 739 (2023).
5. Zuo, C. *et al.* Deep learning in optical metrology: a review. *Light Sci. Appl.* **11**, 39 (2022).
6. Musumeci, F. *et al.* An Overview on Application of Machine Learning Techniques in Optical Networks. *IEEE Commun. Surv. Tutor.* **21**, 1383–1408 (2019).
7. Nevin, J. W. *et al.* Machine learning for optical fiber communication systems: An introduction and overview. *APL Photonics* **6**, 121101 (2021).
8. Agrawal, G. P. *Nonlinear Fiber Optics*. (Elsevier, 2013). doi:10.1016/B978-0-12-397023-7.00018-8.
9. *Supercontinuum Generation in Optical Fibers*. (Cambridge Univ. Press, Cambridge, 2010).
10. Salmela, L. *et al.* Predicting ultrafast nonlinear dynamics in fibre optics with a recurrent neural network. *Nat. Mach. Intell.* **3**, 344–354 (2021).
11. Salmela, L. *et al.* Feed-forward neural network as nonlinear dynamics integrator for supercontinuum generation. *Opt. Lett.* **47**, 802–805 (2022).
12. Teğin, U., Dinç, N. U., Moser, C. & Psaltis, D. Reusability report: Predicting spatiotemporal nonlinear dynamics in multimode fibre optics with a recurrent neural network. *Nat. Mach. Intell.* **3**, 387–391 (2021).
13. Pu, G. *et al.* Fast Predicting the Complex Nonlinear Dynamics of Mode-Locked Fiber Laser by a Recurrent Neural Network with Prior Information Feeding. *Laser Photonics Rev.* **17**, 2200363 (2023).
14. Rudy, S. H., Brunton, S. L., Proctor, J. L. & Kutz, J. N. Data-driven discovery of partial differential equations. *Sci. Adv.* **3**, e1602614 (2017).
15. Jiang, X. *et al.* Physics-Informed Neural Network for Nonlinear Dynamics in Fiber Optics. *Laser Photonics Rev.* **16**, 2100483 (2022).
16. Wetzel, B. *et al.* Customizing supercontinuum generation via on-chip adaptive temporal pulse-splitting. *Nat. Commun.* **9**, 4884 (2018).
17. Wang, Z., Ye, F. & Li, Q. Modified genetic algorithm for high-efficiency dispersive waves emission at 3 μm. *Opt. Express* **30**, 2711–2720 (2022).
18. Hoang, V. T. *et al.* Optimizing supercontinuum spectro-temporal properties by leveraging machine learning towards multi-photon microscopy. *Front. Photonics* **3**, 940902 (2022).
19. Hary, M. *et al.* Tailored supercontinuum generation using genetic algorithm optimized Fourier domain pulse shaping. *Opt. Lett.* **48**, 4512–4515 (2023).
20. Pinto, T. *et al.* Optimization of frequency combs spectral-flatness using evolutionary algorithm. *Opt. Express* **29**, 23447–23460 (2021).
21. Mazoukh, C. *et al.* Genetic algorithm-enhanced microcomb state generation. *Commun. Phys.* **7**, 81 (2024).
22. Andral, U. *et al.* Fiber laser mode locked through an evolutionary algorithm. *Optica* **2**, 275–278 (2015).
23. Pu, G., Yi, L., Zhang, L. & Hu, W. Intelligent programmable mode-locked fiber laser with a human-like algorithm. *Optica* **6**, 362–369 (2019).
24. Meng, F. & Dudley, J. M. Toward a self-driving ultrafast fiber laser. *Light Sci. Appl.* **9**, 26 (2020).
25. Wu, X. *et al.* Intelligent Breathing Soliton Generation in Ultrafast Fiber Lasers. *Laser Photonics Rev.* **16**, 2100191 (2022).

26. Valensise, C. M., Giuseppi, A., Cerullo, G. & Polli, D. Deep reinforcement learning control of white-light continuum generation. *Optica* **8**, 239–242 (2021).
27. Zibar, D. *et al.* Inverse System Design Using Machine Learning: The Raman Amplifier Case. *J. Light. Technol.* **38**, 736–753 (2020).
28. Zhang, C., Kang, G., Wang, J., Pan, Y. & Qu, J. Inverse design of soliton microcomb based on genetic algorithm and deep learning. *Opt. Express* **30**, 44395–44407 (2022).
29. Lucas, E., Yu, S.-P., Briles, T. C., Carlson, D. R. & Papp, S. B. Tailoring microcombs with inverse-designed, meta-dispersion microresonators. *Nat. Photonics* **17**, 943–950 (2023).
30. Iwami, R. *et al.* Controlling chaotic itinerancy in laser dynamics for reinforcement learning. *Sci. Adv.* **8**, eabn8325 (2022).
31. Boscolo, S. & Finot, C. Artificial neural networks for nonlinear pulse shaping in optical fibers. *Opt. Laser Technol.* **131**, 106439 (2020).
32. Zakharov, V. & Shabat, A. Exact theory of two-dimensional self-focusing and one-dimensional self-modulation of waves in nonlinear media. *Sov Phys-JETP* **34**, 62–69 (1972).
33. Ablowitz, M. J. & Segur, H. *Solitons and the Inverse Scattering Transform*. (SIAM, 1981).
34. Novikov, S., Manakov, S. V., Pitaevskii, L. P. & Zakharov, V. E. *Theory of Solitons: The Inverse Scattering Method*. (Springer Science & Business Media, 1984).
35. Akhmediev, N. N. & Korneev, V. I. Modulation instability and periodic solutions of the nonlinear Schrödinger equation. *Theor. Math. Phys.* **69**, 1089–1093 (1986).
36. Trillo, S. & Wabnitz, S. Dynamics of the nonlinear modulational instability in optical fibers. *Opt. Lett.* **16**, 986–988 (1991).
37. Dudley, J. M., Dias, F., Erkintalo, M. & Genty, G. Instabilities, breathers and rogue waves in optics. *Nat. Photonics* **8**, 755–764 (2014).
38. Perego, A. M., Bessin, F. & Mussot, A. Complexity of modulation instability. *Phys. Rev. Res.* **4**, L022057 (2022).
39. Copie, F., Randoux, S. & Suret, P. The physics of the one-dimensional nonlinear Schrödinger equation in fiber optics: Rogue waves, modulation instability and self-focusing phenomena. *Rev. Phys.* **5**, 100037 (2020).
40. Chen, S. *et al.* Modulation instability—rogue wave correspondence hidden in integrable systems. *Commun. Phys.* **5**, 297 (2022).
41. Wabnitz, S. & Wetzel, B. Instability and noise-induced thermalization of Fermi–Pasta–Ulam recurrence in the nonlinear Schrödinger equation. *Phys. Lett. A* **378**, 2750–2756 (2014).
42. Bonetti, J., Hernandez, S. M., Fierens, P. I. & Grosz, D. F. Analytical study of coherence in seeded modulation instability. *Phys. Rev. A* **94**, 033826 (2016).
43. Dylov, D. V. & Fleischer, J. W. Modulation instability of a coherent–incoherent mixture. *Opt. Lett.* **35**, 2149–2151 (2010).
44. Gammaitoni, L., Hänggi, P., Jung, P. & Marchesoni, F. Stochastic resonance. *Rev. Mod. Phys.* **70**, 223–287 (1998).
45. Frisquet, B., Kibler, B. & Millot, G. Collision of Akhmediev Breathers in Nonlinear Fiber Optics. *Phys. Rev. X* **3**, 041032 (2013).
46. Bessin, F. *et al.* Phase-sensitive seeded modulation instability in passive fiber resonators. *Commun. Phys.* **5**, 6 (2022).
47. Nguyen, D. M. *et al.* Incoherent resonant seeding of modulation instability in optical fiber. *Opt. Lett.* **38**, 5338–5341 (2013).
48. Dudley, J. M., Genty, G. & Eggleton, B. J. Harnessing and control of optical rogue waves in supercontinuum generation. *Opt. Express* **16**, 3644–3651 (2008).
49. Bendahmane, A., Fatome, J., Finot, C., Millot, G. & Kibler, B. Coherent and incoherent seeding of dissipative modulation instability in a nonlinear fiber ring cavity. *Opt. Lett.* **42**, 251–254 (2017).
50. Mussot, A., Conforti, M., Trillo, S., Copie, F. & Kudlinski, A. Modulation instability in dispersion oscillating fibers. *Adv. Opt. Photonics* **10**, 1–42 (2018).

51. Sheveleva, A., Colman, P., Dudley, J. M. & Finot, C. Phase space topology of four-wave mixing reconstructed by a neural network. *Opt. Lett.* **47**, 6317–6320 (2022).
52. Ermolaev, A. V., Sheveleva, A., Genty, G., Finot, C. & Dudley, J. M. Data-driven model discovery of ideal four-wave mixing in nonlinear fibre optics. *Sci. Rep.* **12**, 12711 (2022).
53. Kalmykov, N. I., Zagidullin, R., Rogov, O. Y., Rykovanov, S. & Dylov, D. V. Suppressing modulation instability with reinforcement learning. *Chaos, Solitons & Fractals* **186**, 115197 (2024).
54. Solli, D. R., Herink, G., Jalali, B. & Ropers, C. Fluctuations and correlations in modulation instability. *Nat. Photonics* **6**, 463–468 (2012).
55. Godin, T. *et al.* Recent advances on time-stretch dispersive Fourier transform and its applications. *Adv. Phys. X* **7**, 2067487 (2022).
56. Närhi, M. *et al.* Real-time measurements of spontaneous breathers and rogue wave events in optical fibre modulation instability. *Nat. Commun.* **7**, 13675 (2016).
57. Kraych, A. E., Agafontsev, D., Randoux, S. & Suret, P. Statistical Properties of the Nonlinear Stage of Modulation Instability in Fiber Optics. *Phys. Rev. Lett.* **123**, 093902 (2019).
58. Sader, L. *et al.* Single-Photon Level Dispersive Fourier Transform: Ultrasensitive Characterization of Noise-Driven Nonlinear Dynamics. *ACS Photonics* **10**, 3915–3928 (2023).
59. Närhi, M. *et al.* Machine learning analysis of extreme events in optical fibre modulation instability. *Nat. Commun.* **9**, 4923 (2018).
60. Salmela, L., Lapre, C., Dudley, J. M. & Genty, G. Machine learning analysis of rogue solitons in supercontinuum generation. *Sci. Rep.* **10**, 9596 (2020).
61. Mabed, M. *et al.* Neural network analysis of unstable temporal intensity peaks in continuous wave modulation instability. *Opt. Commun.* 129570 (2023) doi:10.1016/j.optcom.2023.129570.
62. Coulibaly, S., Bessin, F., Clerc, M. G. & Mussot, A. Precursors-driven machine learning prediction of chaotic extreme pulses in Kerr resonators. *Chaos, Solitons & Fractals* **160**, 112199 (2022).
63. Eeltink, D. *et al.* Nonlinear wave evolution with data-driven breaking. *Nat. Commun.* **13**, 2343 (2022).
